# Effect of different γ-subunit isoforms on the regulation of AMPK

**DOI:** 10.1042/BCJ20170046

**Published:** 2017-05-09

**Authors:** Robin Willows, Naveenan Navaratnam, Ana Lima, Jon Read, David Carling

**Affiliations:** 1 Cellular Stress Group, Medical Research Council London Institute of Medical Sciences, Hammersmith Hospital, Imperial College, London W12 0NN, U.K.; 2 AstraZeneca R&D, Discovery Sciences, Darwin Building, 310 Cambridge Science Park, Milton Road, Cambridge CB4 0WG, U.K.; 3 Institute of Clinical Sciences, Faculty of Medicine, Imperial College London, Du Cane Road, London W12 0NN, U.K.

**Keywords:** AMPK, metabolic regulation, protein–serine–threonine kinases

## Abstract

AMP-activated protein kinase (AMPK) plays a key role in integrating metabolic pathways in response to energy demand. AMPK activation results in a wide range of downstream responses, many of which are associated with improved metabolic outcome, making AMPK an attractive target for the treatment of metabolic diseases. AMPK is a heterotrimeric complex consisting of a catalytic subunit (α) and two regulatory subunits (β and γ). The γ-subunit harbours the nucleotide-binding sites and plays an important role in AMPK regulation in response to cellular energy levels. In mammals, there are three isoforms of the γ-subunit and these respond differently to regulation by nucleotides, but there is limited information regarding their role in activation by small molecules. Here, we determined the effect of different γ-isoforms on AMPK by a direct activator, 991. In cells, 991 led to a greater activation of γ2-containing AMPK complexes compared with either γ1 or γ3. This effect was dependent on the long N-terminal region of the γ2-isoform. We were able to rule out an effect of Ser^108^ phosphorylation, since mutation of Ser^108^ to alanine in the β2-isoform had no effect on activation of AMPK by 991 in either γ1- or γ2-complexes. The rate of dephosphorylation of Thr^172^ was slower for γ2- compared with γ1-complexes, both in the absence and presence of 991. Our studies show that activation of AMPK by 991 depends on the nature of the γ-isoform. This finding may have implications for the design of isoform-selective AMPK activators.

## Introduction

AMP-activated protein kinase (AMPK) is a highly conserved heterotrimeric serine/threonine kinase widely characterised as a sensor of cellular energetic stress [1,2]. In response to activation by a decrease in ATP levels, AMPK phosphorylates many downstream targets involved in a diverse range of metabolic pathways [1,3,4]. The overall effect of AMPK activation is to turn off anabolic, ATP-utilising pathways and turn on catabolic, ATP-generating pathways. For instance, AMPK phosphorylates and inactivates acetyl-CoA carboxylase (ACC), which leads to inhibition of fatty acid synthesis and/or activation of fatty acid oxidation [1]. AMPK is a heterotrimeric complex consisting of a catalytic α-subunit and two regulatory subunits (β and γ) [1,5]. In mammals, there are two α-subunit isoforms, together with two β- and three γ-isoforms, leading to 12 different possible AMPK complexes. The γ-subunit possesses four cystathionine-β-synthase (CBS) domains, and these are involved in binding adenine nucleotides [5–7]. AMP binding to AMPK results in allosteric activation, which when assayed in the presence of physiological concentrations of ATP (5 mM) can reach up to 10-fold [8]. AMPK is activated 100–1000-fold following phosphorylation of the activation loop residue, Thr^172^ (threonine 172 in the rat α-subunit), by upstream kinases, including LKB1 (liver kinase B1) and calcium/calmodulin-dependent protein kinase kinase-β [9–14]. A number of studies have reported that nucleotides regulate phosphorylation of AMPK by the upstream kinases, although it is not clear whether both AMP and ADP, or AMP alone, mediate this effect [8,15,16]. More recently, AMP has been shown to promote binding of AMPK and LKB1 to axin, providing a potential mechanism for increased AMPK phosphorylation by AMP [17]. In addition to these effects, both AMP and ADP protect against dephosphorylation of Thr^172^ [7,18–20]. Two recent studies reported that the effects of nucleotides on AMPK activity depend on the isoform composition of the AMPK complexes [8,21].

Given its central role in regulating energy homeostasis, systemic activation of AMPK provides an attractive pharmacological target for combatting metabolic diseases, and many AMPK activators have been reported [22–24]. Most of the small-molecule AMPK activators that have been identified to date, such as A769662 and 991, bind to a single site formed between the α- and β-subunits [24,25]. These direct activators protect against Thr^172^ dephosphorylation and allosterically activate AMPK, although the mechanism by which they activate AMPK is distinct from the nucleotide-mediated effects on AMPK [24,25]. Similar to AMP, allosteric activation by synthetic small molecules depends on the isoform composition of the AMPK complex [21,24]. In addition, binding is significantly tighter with β1- versus β2-containing AMPK complexes, and recently, a highly selective β1-activator was reported [22]. An important consideration in the development of AMPK activators as therapeutic drugs is the potential for deleterious effects of chronic AMPK activation *in vivo*. This is a particular concern with AMPK since dominantly inherited gain-of-function mutations in the γ2-subunit of AMPK have been identified in humans [26]. These mutations lead to a cluster of cardiac abnormalities, including Wolff–Parkinson–White syndrome and cardiac hypertrophy associated with glycogen accumulation [27–29]. A recent study has also reported that humans carrying the R302Q mutation in γ2 have increased adiposity and slightly raised fasting glucose levels compared with unaffected individuals [30]. This raises a concern for potential detrimental effects of chronic, systemic AMPK activation, although to date there have been no published studies for long-term *in vivo* effects of specific AMPK activators. Determining the effects of pharmacological activators on different AMPK complexes, and understanding whether there are any differences in their mechanism of activation, especially for γ2-containing AMPK complexes, is an important factor in the design and development of potential drugs. Currently, there is little information regarding the effect of AMPK activators on γ2-complexes, and in the recent work by Rajamohan et al. [21], γ2-containing AMPK complexes were not included in the study.

In this study, we investigate the response of different γ-isoform AMPK complexes to pharmacological stimulation by 991, a potent direct activator of AMPK [24]. We show that in cell-based assays, γ2-complexes are activated by 991 to a greater extent than either γ1- or γ3-complexes. This effect is mediated by the N-terminal region of γ2 and is probably due to enhanced protection of Thr^172^ from dephosphorylation. These findings suggest a role for the long N-terminal region of γ2 in regulating AMPK activity that has not previously been recognised. Our results may also have important implications for the design and development of AMPK activators aimed at therapeutic intervention in human disease states.

## Experimental

### Materials and proteins

991 was synthesised as described previously [24]. PF06685249 [31] was a generous gift from Dr Russell Miller (Pfizer Global Research and Development). Recombinant PP2Cα was purified as described previously [32].

### Cell culture

HEK293T cells were maintained in Dulbecco's Modified Eagle's Medium (DMEM; Thermo) supplemented with 10% foetal bovine serum (Sigma–Aldrich). AMPKα, -β and -γ constructs were cloned into pcDNA3 (Invitrogen) for transient expression, or pLPC (Addgene) with a puromycin selection cassette for stable transfection. For transfection experiments, cells were plated on 6 cm plates at 75% density and transfected using polyethylenimine (PEI; Polysciences) reagent at 2 μg per construct with a PEI:DNA ratio of 3:1 (w/w). Thirty-six hours post-transfection, cells were transferred to serum-free DMEM for 2 h prior to treatment with 991 or PF249 (for 30 min). As a vehicle control, DMSO was added at the same concentration as for the 991-treated cells. Cells were washed three times with ice-cold PBS before the addition of lysis buffer: 50 mM HEPES (pH 7.4), 50 mM sodium fluoride, 5 mM sodium pyrophosphate, 1 mM ethylenediaminetetraacetic acid, 10% (v/v) glycerol, 1% (v/v) Triton X-100, 1 mM dithiothreitol, 0.1 mM phenylmethylsulfonyl fluoride, 4 μg/ml trypsin inhibitor and 0.1 mM benzamidine. For stable transfections, subunits were cloned into pLPC vector and selected 48 h post-transfection with 3 μg/ml puromycin (Thermo). Cell lines were maintained in puromycin and plated at 80% confluency the day before serum starvation and treatment as described above.

### CRISPR-mediated deletion of β-subunit isoforms

HEK293T cells were transfected with plasmids containing Cas9 linked to GFP expression via a self-cleaving peptide and guide sequences targeting the first exon of β1 (GCTGGTATTGCCCATGATGG, GCCACCATGCCGCTCCAGCG, GGGCTGTCCATCAGGATCTT, TTCCTCGGAGTGGAAGAGGT, TCAAGGTGCGAGCGGTGTGG) or β2 (CCCATGGCTGCAGCTCGTCG, ACCACCAGCGACCGGGTGTC, AGCGTGCAGCCTTGGCGCCG, ATGATCTTGTGCTCCTTCCC, CAGGGAGGCTGAACACGCTG) (Horizon Discovery, Cambridge, U.K.). Twenty-four hours post-transfection, cells were sorted based on GFP expression and individual colonies were analysed by western blotting.

### Western blotting

The protein concentration of cell lysates was determined by protein assay (Bio-Rad) prior to heating for 5 min at 95°C in 5× SDS loading buffer. Proteins (15 μg total) were resolved by SDS–PAGE on 10% polyacrylamide gels (National Diagnostics) and transferred to Immobilon-FL (Millipore) membranes at 4°C. Membranes were probed with primary antibodies at a 1:1000 dilution and incubated overnight at 4°C. The following antibodies were from Cell Signalling: mouse anti-α1/2 (#2793), rabbit anti-β1/2 (#4150), rabbit anti-γ1 (#4187), rabbit anti-His (#2365) and rabbit anti-pThr^172^ (#2535). Rabbit anti-GAPDH was from Abcam (9485); mouse anti-vinculin (Sigma–Aldrich, V9131); mouse anti-FLAG M2 (Stratagene, 200472). After extensive washing, membranes were incubated for 30–60 min with LI-COR secondary antibodies diluted 1:10 000. Blots were imaged on an LI-COR Odyssey CLX. Peak area ratios for pThr^172^ to total α1/2 were obtained using Image Studio (LI-COR) to determine fold activation profiles. For capillary western blotting, cell lysates were diluted in HEPES lysis buffer to 0.6 mg/ml for experiments conducted on endogenous protein, or 0.1 mg/ml for experiments using transfected cells. Samples were prepared and analysed according to the manufacturer's instructions (ProteinSimple). Rabbit anti-β1/2, anti-pThr^172^ and anti-pACC (Cell Signalling, 3661) were all used at 1:200 dilution in antibody dilution buffer (ProteinSimple).

### Immunoprecipitation of AMPK complexes

To obtain purified complexes for *in vitro* allosteric and protection assays, β1β2 double knockout (dKO) cells were transfected as described above and treated with 5 μM 991 (30 min) prior to lysis. FLAG-tagged complexes were immunoprecipitated by incubating lysate supernatant with FLAG M2 affinity gel (Sigma–Aldrich, A2220) overnight with gentle agitation. Following extensive washing with HEPES lysis buffer containing 300 mM NaCl, the resin was washed twice with 50 mM HEPES buffer (pH 7.4). Complexes were eluted with 2 × 50 μl of FLAG peptide (Sigma–Aldrich, F3290; 5 μg/ml) in this buffer.

### AMPK assay

AMPK was assayed using the SAMS peptide assay as described previously [33]. Briefly, purified FLAG-tagged AMPK was incubated with 0.2 mM SAMS peptide, in the absence or presence of varying concentrations of 991 [in all cases, the final concentration of DMSO was kept at 0.5% (v/v)]. Incorporation of radiolabelled phosphate from ATP into SAMS peptide was measured by liquid scintillation counting. Isolated complexes were titrated to obtain a similar basal AMPK activity (measured in the absence of 991).

### Protection against dephosphorylation

Purified AMPK complexes were dephosphorylated by incubating at 37°C with 2.5 μg/ml PP2C and 2.5 mM MgCl_2_ in the absence or presence of 100 nM 991. Reactions were stopped by dilution (1:10) into ice-cold 50 mM HEPES buffer (pH 7.4), containing 50 mM NaF and 5 mM sodium pyrophosphate. AMPK activity was determined using the SAMS assay.

### Statistical analysis

Where appropriate, results were analysed using either Student's *t*-test or ANOVA using Bonferroni correction.

## Results

### Increased 991-stimulated Thr^172^ phosphorylation in AMPKγ2 complexes compared with AMPKγ1

**Figure 1.**
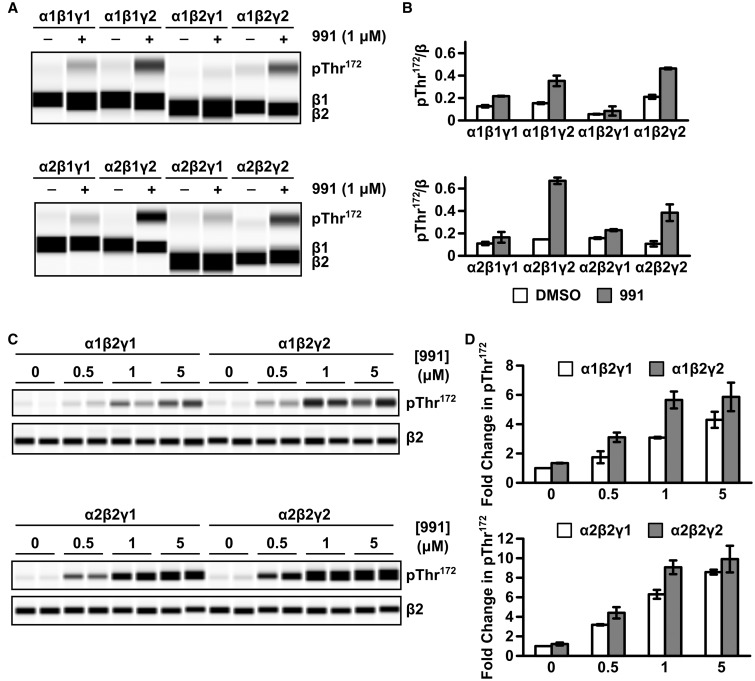
Activation of γ1 versus γ2 AMPK complexes by 991. HEK293T cells were co-transfected with cDNA encoding either γ1 or γ2 together with α1 or α2 and β1 or β2. Thirty-six hours post-transfection, cells were serum-starved for 2 h and incubated with DMSO or 991 (1 μM) for 30 min prior to cell lysis. (**A**) Representative blots showing Thr^172^ phosphorylation and β1/β2 expression, determined by western blotting using an automated capillary-based electrophoresis system (Simon, Protein Simple), are shown. (**B**) Graphs showing quantification of Thr^172^ phosphorylation relative to β-subunit expression. (**C**) Thr^172^ phosphorylation and β2 expression were determined in response to varying concentrations of 991. (**D**) Fold change in Thr^172^ phosphorylation: β-subunit expression relative to the corresponding γ1-complex in the absence of 991 is shown. Results shown for (**B**) and (**D**) are mean ± SEM for two independent experiments.

Two recent studies examined the role of AMPK subunit isoforms in regulation by nucleotides and pharmacological activators [8,21]. Despite these studies, however, the regulation of γ2-containing AMPK complexes by pharmacological activators was not investigated. AMPKγ2 is widely expressed in mammalian tissues and mutations in *PRKAG2*, the gene encoding γ2, cause a cluster of severe cardiac abnormalities in humans [27–29]. The lack of information regarding the regulation of AMPKγ2 complexes by pharmacological activators prompted us to investigate this further. We have been unable to express recombinant AMPKγ2 complexes in *Escherichia coli*, and hence we used mammalian expression for our study. We co-expressed γ1 or γ2 (isoform γ2a, corresponding to the long form of γ2 [34]) with either α1/α2 or β1/β2 in HEK293T cells and measured Thr^172^ phosphorylation of the different AMPK complexes in response to activation by 991 (1 μM), a potent pharmacological AMPK activator [24]. In all cases, there was a marked increase in 991-stimulated Thr^172^ phosphorylation with the γ2-containing AMPK complexes compared with the corresponding γ1-containing complexes (Figure 1A,B). We carried out a similar experiment, but using varying concentrations of 991 (Figure 1C,D). In this experiment, we restricted our analysis to complexes containing the β2-isoform, as this isoform is the predominant β-subunit isoform expressed in the heart [35]. Consistent with our initial experiment, 991-stimulated Thr^172^ phosphorylation was greater in complexes containing the γ2-isoform relative to the γ1-isoform. There was also a trend for increased Thr^172^ phosphorylation in the γ2- versus γ1-complexes in the absence of 991, although this was not statistically significant. The increase in 991-induced Thr^172^ phosphorylation was similar for both α1 and α2 AMPK complexes, and since α2 is the predominant α-isoform expressed in heart [36], we focussed on α2β2γ1- and α2β2γ2-complexes for our subsequent studies.

### CRISPR-generated β1/2 knockout HEK293T cell line

**Figure 2.**
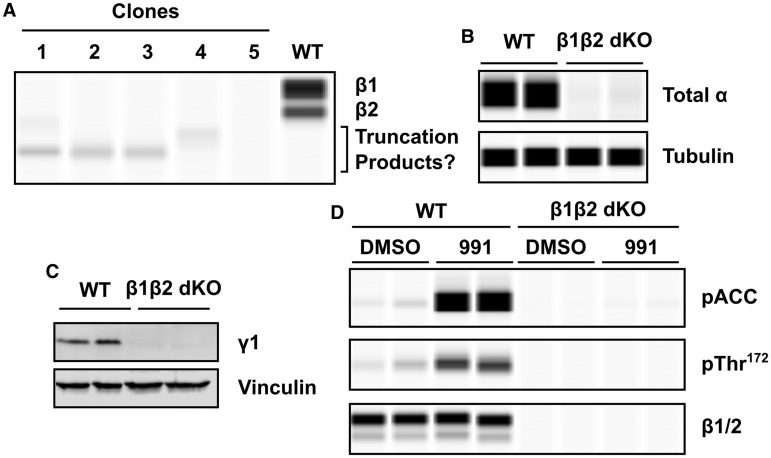
Generation of β1β2 dKO cells. Clones of HEK293T cells were isolated by flow cytometry following transfection with plasmids containing Cas9 and guide RNAs targeting the β1- and β2-isoforms of AMPK. (**A**) A representative capillary western blot of β subunit expression in five different clones compared with wild-type HEK293T is shown. Lower molecular mass cross-reacting bands, potentially due to truncated β-subunit protein, are indicated. (**B**) Total AMPKα and (**C**) AMPKγ1 expression in β1β2 dKO cells relative to wild-type cells were determined by western blotting. (**D**) Wild-type and β1β2 dKO cells were incubated with DMSO or 991 (1 μM) for 30 min. Levels of ACC and Thr^172^ phosphorylation, together with β-subunit expression, were determined. In each case, a representative blot with two independent samples is shown.

A potential complication with studying isoform-specific roles of AMPK subunits using mammalian expression systems is that the endogenous AMPK complexes could interfere with subsequent analyses. To circumvent this potential issue, we generated HEK293T cells lacking functional AMPK complexes. We used the CRISPR–Cas9 system to knockout expression of AMPKβ1 and -β2. We chose to delete the β-subunits as these isoforms have been shown previously to play an important role in compound binding at the kinase domain–carbohydrate binding module (CBM) interface. In addition, there is a highly specific and sensitive antibody raised to a sequence that is conserved in both β1 and β2 that allows for detection of these isoforms (although the predicted masses of β1 and β2 are very similar, they are readily resolved by SDS–PAGE [35]). Cells were first transfected with guides targeting the first exon of β1 and clones lacking expression of β1 (as assessed by western blotting) were isolated. Clones lacking detectable β1 expression were subsequently transfected with guides targeting the first exon of β2, and cells lacking detectable β2 expression were identified by blotting. In most cases, we observed clones that, although lacking expression of full-length β1 and β2, appeared to express truncated forms of the β-subunit that cross-reacted with the anti-β antibody (Figure 2A). However, in some clones, there was no detectable cross-reactivity (e.g. clone 5 in Figure 2A). Clones lacking detectable β-subunit expression, which we term β1β2 dKO cells, were expanded and used for subsequent studies of AMPK regulation.

Total AMPKα and AMPKγ1 expression were barely detectable in the β1β2 dKO cells (Figure 2B,C). We have been unable to detect endogenous γ2 or γ3 expression in wild-type HEK293T cells using either commercial or in-house antibodies and hence cannot determine the effect of deletion of the β-subunit on their expression. However, a previous study reported that γ1 is the predominant γ-isoform in HEK293 cells [37]. Moreover, phosphorylation of Thr^172^ and ACC, a widely used downstream substrate for AMPK, were barely detectable in the β1β2 dKO cells, either in the absence or presence of 991 (Figure 2C). In contrast, phosphorylation of both Thr^172^ and ACC was readily detected in wild-type cells and markedly increased following treatment with 991. Taken together, these findings demonstrate that CRISPR/Cas9-mediated deletion of β1 and β2 in HEK293T cells generates a cell line almost completely devoid of functional AMPKs.

**Figure 3.**
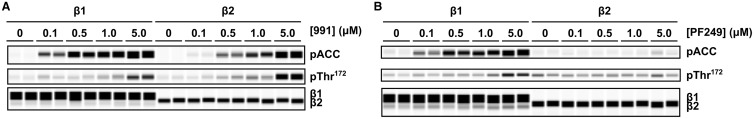
Activation of β1- and β2-AMPK complexes by direct small-molecule activators. AMPKβ1 or AMPKβ2 expressing stable cell lines were generated in the β1β2 dKO HEK293T cells. Cells were incubated with different concentrations of 991 (**A**) or PF249 (**B**). Levels of ACC and Thr^172^ phosphorylation, together with β-subunit expression, were determined.

Next, we generated cell lines in which we stably reintroduced either β1 or β2 into the β1β2 dKO cells. This provided us with cells with defined β-isoform expression, allowing us to investigate β-isoform-specific regulation of AMPK. Cells were treated in the absence or presence of increasing concentrations of two different AMPK activators, 991 or PF06685249 (hereafter referred to as PF249), a β1-selective AMPK activator [31]. In both β1- and β2-expressing cells, 991 caused a dose-dependent increase in Thr^172^ and ACC phosphorylation (Figure 3A). We previously reported that β1-complexes bound 991 with ∼10-fold higher affinity compared with β2-complexes [24]. Consistent with this, we observed that phosphorylation of ACC is more sensitive to 991 in β1-expressing cells relative to β2-expressing cells. It is also clear that the 991-induced increase in ACC phosphorylation is markedly more pronounced than that in Thr^172^ phosphorylation, particularly at low concentrations. This presumably reflects the fact that, in addition to increasing Thr^172^ phosphorylation, 991 causes a significant allosteric activation of AMPK, which would result in more marked phosphorylation of downstream targets. Treatment with PF249, which is more than 1000-fold more potent towards β1- compared with β2-complexes [31], led to a robust increase in ACC phosphorylation, together with a weaker, but detectable, increase in Thr^172^ phosphorylation in β1-expressing cells. Importantly, however, PF249 had virtually no effect on Thr^172^ or ACC phosphorylation in β2-expressing cells (Figure 3B). There was a very slight increase in ACC phosphorylation in the β2-expressing cells at the highest concentration of 991 (5 μM), but this was less than the phosphorylation of ACC observed at the lowest concentration (0.1 μM) in the β1-expressing cells. These results confirm that AMPK signalling is restored by re-expression of the β-subunit in the β1β2 dKO cells, and demonstrate the β-isoform selectivity of AMPK activators in human cells.

### γ2 N-terminal region mediates the increased 991-induced Thr^172^ phosphorylation

**Figure 4.**
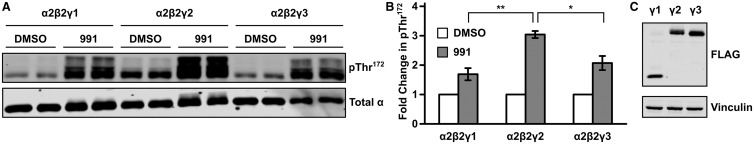
Differential activation of AMPKγ isoforms by 991. FLAG-tagged AMPKγ1, -γ2 and -γ3 constructs were transiently co-transfected with α2 and β2 into β1β2 dKO cells. Cells were treated with or without 991 (1 μM) for 30 min. (**A**) Thr^172^ phosphorylation and total α expression levels were determined by fluorescence-based western blotting. (**B**) Blots were quantified and the increase in Thr^172^ phosphorylation in response to 991 relative to the DMSO control was determined as a fold change. Results are for three independent experiments, performed in duplicate, and are plotted as means ± SEM. Statistically significant results, determined by one-way ANOVA, using Bonferroni correction, are indicated: *P < 0.05, **P < 0.01. There was no significant difference between γ1- and γ3-complexes. (**C**) Expression of the different γ- isoforms in the AMPK complex was confirmed by western blotting using an anti-FLAG antibody.

**Figure 5.**
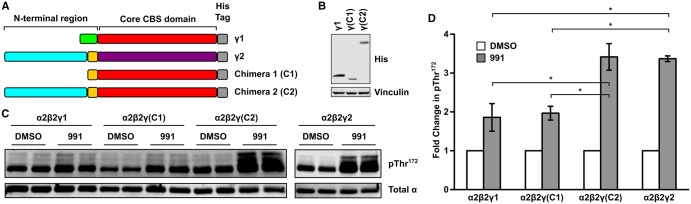
Increased activation by 991 is mediated by the N-terminal region of γ2. (**A**) A schematic representation of the γ1- and γ2-isoforms is shown, together with the two chimeric γ-constructs [γ(C1) and γ(C2)] used in the present study. Different regions within the isoforms are shown colour-coded. The conserved core region, containing the four CBS repeats, is indicated in red for γ1 and purple for γ2. The N-terminal region of γ1 is shown in green, the N-terminal sequence for γ2b (the short isoform of γ2) is shown in yellow and the long N-terminal extension in γ2a (the long isoform of γ2) is coloured in blue. All constructs have a C-terminal hexa-His tag shown in grey. (**B**) Expression of the two chimeric proteins was confirmed by western blotting using an anti-His antibody and is shown relative to γ1-expression. Vinculin expression was used as a loading control. (**C**) β1β1 dKO cells were transfected with different AMPK complexes and treated with DMSO or 991 (1 μM) for 30 min. Thr^172^ phosphorylation and total α expression levels were determined by fluorescence-based western blotting. Although the α2β2γ2-complex was analysed in the same experiment at the same time, it was performed on a separate blot and is therefore shown as a distinct panel. (**D**) Blots were quantified and the increase in Thr^172^ phosphorylation in response to 991 relative to the DMSO control was determined as a fold change. Results are for three independent experiments, performed in duplicate, and are plotted as means ± SEM. Statistically significant results, determined by one-way ANOVA, using Bonferroni correction, are indicated (*P < 0.05).

The γ-isoforms all contain a highly conserved C-terminal region harbouring the four CBS domains involved in nucleotide binding. In addition, the γ2- and γ3-isoforms contain long N-terminal extensions that are not present in the γ1-isoform [38]. These N-terminal extensions show no obvious sequence conservation between isoforms, or significant homology with other proteins. In addition, variant forms of γ2- and γ3-transcripts have been identified that encode predicted proteins with truncated N-terminal regions [34,39]. At present, it is not known whether the N-terminal extensions of γ2 or γ3 play any specific functional role, or what role, if any, the different variants play *in vivo*. To investigate further the effect of the different γ-isoforms on AMPK regulation, we expressed α2β2 with either γ1, γ2 or γ3 in the β1β2 dKO cells and compared Thr^172^ in the different AMPK complexes in response to 991. As shown in Figure 4, 991 stimulated Thr^172^ phosphorylation of all AMPK complexes. However, the increase in Thr^172^ phosphorylation within the γ2-complex was significantly greater than that in either the γ1- or γ3-complexes. To determine whether the enhanced response of γ2-containing complexes to 991 was mediated by its N-terminal region, we synthesised constructs in which we replaced the N-terminal region of γ1 (corresponding to the first 31 amino acids of γ1) with sequences from γ2 (see Figure 5A). In one chimeric construct (C1), we replaced the γ1 sequence with the N-terminal sequence of the short form of γ2 (termed γ2b), and in a second construct (C2), we replaced it with the N-terminal sequence of the long form of γ2 (termed γ2a). It is important to note that despite numerous attempts, and using a variety of epitope tags at both the N- and C-termini, we have been unable to express the short form of γ2 (isoform γ2b) in mammalian cells. In addition, we were unable to express a chimeric construct in which we replaced the N-terminal sequence of γ2a with the N-terminal region of γ1 (data not shown). We co-expressed the two chimeric γ-constructs with α2 and β2 and confirmed their expression by western blotting with an anti-His antibody directed against a C-terminal hexa-histidine tag included in the constructs (Figure 5B). Thr^172^ phosphorylation was markedly enhanced in the AMPK complex containing chimera 2 (which has the entire N-terminal region of γ2a replacing the N-terminal region of γ1) and was similar to Thr^172^ phosphorylation in AMPK complexes containing native γ2 (Figure 5C,D). In contrast, Thr^172^ phosphorylation of AMPK containing chimera 1 (replacing the N-terminal region of γ1 with the short N-terminal region of γ2b) was the same as in AMPK containing native γ1. These data strongly suggest that the enhanced response of γ2-AMPK to 991 is mediated by the long N-terminal region of γ2.

### Phosphorylation of Ser^108^ in β2

**Figure 6.**
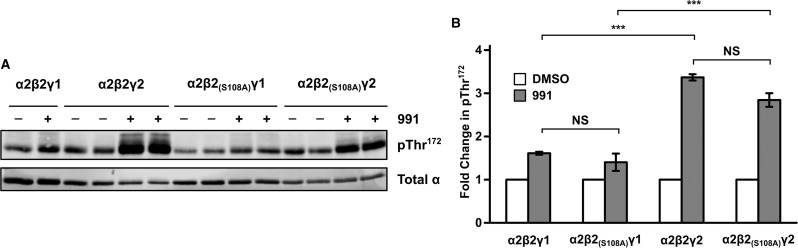
Mutation of Ser^108^ in β2 does not mediate activation by 991 in cells. β1β2 dKO cells were transfected with α2 and either γ1 or γ2 with either wild-type β2 or β2 harbouring mutation of Ser^108^ to alanine (β2_(S108A)_). Cells were treated with DMSO or 991 (1 μM) for 30 min and analysed for Thr^172^ phosphorylation and total α expression, and a representative western blot is shown in (**A**). (**B**) Blots were quantified and the increase in Thr^172^ phosphorylation in response to 991 relative to the DMSO control was determined as a fold change. Results are for three independent experiments, performed in duplicate, and are plotted as means ± SEM. Statistically significant results, determined by one-way ANOVA, using Bonferroni correction, are indicated (***P < 0.001). There was no statistically significant difference (NS) between wild-type β2 and β2_(S108A)_ for either γ1- or γ2-complexes.

**Figure 7.**
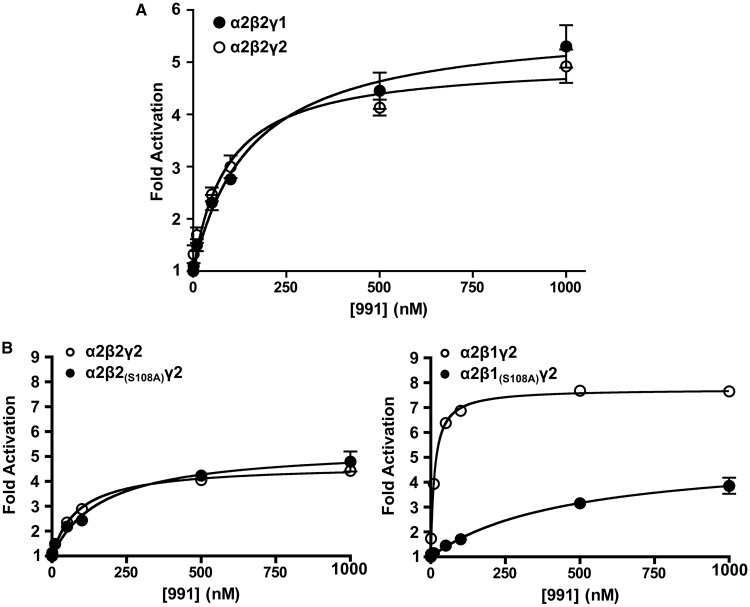
Allosteric activation of AMPKγ1 and -γ2 complexes by 991. AMPK α2β2γ1- and α2β2γ2-complexes (harbouring a FLAG tag at the N-terminus of the γ-isoform) were expressed in β1β2 dKO cells and isolated by immunoprecipitation using FLAG affinity resin. After extensive washing, proteins were eluted using FLAG peptide. (**A**) AMPK activity was measured using the SAMS peptide assay in the presence of varying concentrations of 991 (*n* = 5 independent experiments). (**B**) AMPK α2β1γ2, α2β1_(S108A)_γ2, α2β2γ2 and α2β2_(S108A)_γ2 were expressed in β1β2 dKO cells and isolated by immunoprecipitation using FLAG affinity resin (the β-constructs contain a C-terminal FLAG tag). AMPK activity for each of the complexes was measured using the SAMS peptide assay in the presence of varying concentrations of 991 (*n* = 3 independent experiments). The data were fitted to the equation: *v* = *b* + {[(*s* × *b*) − *b*] × [991]}/(*A*_0.5_ + [991]), where *s* is the relative stimulation, *b* is the basal activity and *A*_0.5_ is the concentration of 991 giving half-maximal stimulation, using the GraphPad Prism software. In all cases, results are plotted as the means ± SEM and the theoretical curves were fitted using the curve-fitting programme.

Previous studies have shown that phosphorylation of serine 108 (Ser^108^) in the β1-subunit significantly increases the binding affinity of AMPK to both 991 and A769662 [24], leading us to speculate whether Ser^108^ phosphorylation might play a role in the enhanced activation of γ2-containing AMPK complexes by 991. Moreover, since our experimental system focussed mainly on β2-containing AMPK complexes, this also provided us with an opportunity to examine the role of Ser^108^ phosphorylation in β2, which has not been reported previously. We expressed β2 harbouring mutation of Ser^108^ to a non-phosphorylatable alanine β2_(S108A)_ with either α2γ1 or α2γ2 in β1β2 dKO cells. As shown in Figure 6, Thr^172^ phosphorylation in response to treatment with 991 was similar in AMPK complexes containing either wild-type β2 or β2_(S108A)_. This contrasts with the effect of S108A mutation in β1, where activation by 991 is significantly reduced in AMPK complexes harbouring β1_(S108A)_ compared with AMPK containing wild-type β1 [24]. The lack of effect of the β2_(S108A)_ mutation on AMPK activation in cells is intriguing, as the region surrounding Ser^108^ shows a high degree of amino acid sequence conservation between β1 and β2 [35]. We extended our study to determine the effect of the S108A mutation in β2 on allosteric activation of AMPK by 991 in cell-free assays (Figure 7 and Table 1). AMPK complexes were expressed in HEK293T cells and isolated by affinity purification using FLAG resin. AMPK activity was determined in the absence or presence of varying concentrations of 991. Wild-type α2β2γ1- and α2β2γ2-complexes were allosterically activated ∼5-fold and showed very similar dose–response curves with 991 (Figure 7A). There was no significant difference in the *A*_0.5_ for 991 between the γ1- and γ2-containing complexes (Table 1), indicating that 991 binds with similar affinity to both complexes. Consistent with the cell-based studies, there was no significant difference in allosteric activation of AMPK containing wild-type β2 versus β2 harbouring the S108A mutation (Figure 7B). In marked contrast, the S108A mutation in β1 caused a marked rightward shift in the dose–response curve for 991, similar to the effect we previously reported for AMPKβ1 complexes expressed in *E. coli* [24]. These findings suggest that, unlike β1, phosphorylation of Ser^108^ in β2 does not play a significant role in binding or activation of AMPK by 991. Moreover, in keeping with our previous study using bacterially expressed AMPK complexes, 991 was more potent and caused a greater level of allosteric activation (8-fold versus 5-fold), with mammalian expressed β1-containing AMPK complexes compared with β2 complexes (Figure 7B and Table 1).

**Table 1:** Allosteric activation of AMPK complexes by 991 AMPK complexes expressed in β1β2 dKO cells were isolated by immunoprecipitation using FLAG affinity resin. After extensive washing, proteins were eluted using the FLAG peptide and AMPK activity was measured using the SAMS peptide assay in the presence of varying concentrations of 991. The data were fitted to the equation: *v* = *b* + {[(*s* × *b*) − *b*] × [991]}/(*A*_0.5_ + [991]), where *s* is the relative stimulation, *b* is the basal activity and *A*_0.5_ is the concentration of 991 giving half-maximal stimulation, using the GraphPad Prism software. In all cases, results are from at least three independent experiments and are plotted as the means ± SEM.

Complex	*A*_0.5_ 991 (nM)	Activation (fold)
α2β2γ1	147 ± 28	5.7 ± 0.3
α2β2γ2	97 ± 18	5.0 ± 0.2
α2β2_(S108A)_γ2	170 ± 35	5.4 ± 0.3
α2β1γ2	12.7 ± 4	7.7 ± 0.4
α2β1_(S108A)_γ2	462 ± 142	5.2 ± 0.5

### Effect of the γ-isoform on protection against Thr^172^ dephosphorylation

**Figure 8.**
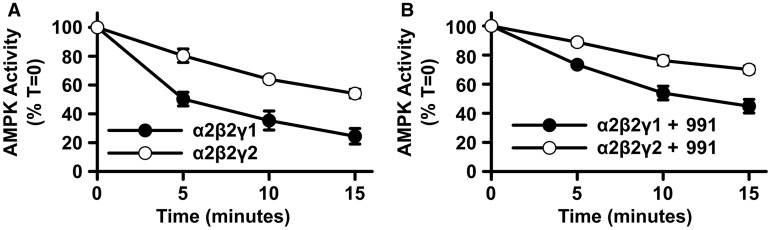
Protection against dephosphorylation of AMPKγ1 and -γ2 complexes by 991. AMPK α2β2γ1- and α2β2γ2-complexes (harbouring a FLAG tag at the N-terminus of the γ-isoform) were expressed in β1β2 dKO cells and purified using FLAG affinity resin. AMPK complexes were incubated with recombinant PP2Cα in the absence (**A**) or presence of 100 nM 991 (**B**). At the indicated times, aliquots were removed and diluted in buffer containing protein phosphatase inhibitors, and AMPK activity was measured using the SAMS peptide assay. The amount of AMPK was adjusted so that an equal amount of AMPK activity was added at the start of the incubation (*T* = 0). Results are plotted as the percentage of activity remaining relative to the starting activity at time 0. In both cases, results are from five independent experiments and are the means ± SEM.

The finding that the γ1 and γ2 AMPK complexes show similar allosteric activation by 991 indicates that there is no difference in binding between the complexes. This rules out the possibility that the increased 991-induced Thr^172^ phosphorylation of the γ2-complex is due to increased affinity for 991. We therefore decided to investigate whether the γ-isoforms might play a role in protection against Thr^172^ dephosphorylation. Recombinant AMPK complexes were purified from HEK293T cells and the rate of dephosphorylation by PP2C was measured in the absence (Figure 8A) or presence (Figure 8B) of 991. Consistent with previous studies, 991 protected AMPK against Thr^172^ dephosphorylation for both γ1- and γ2-complexes. Importantly, however, the rate of dephosphorylation of γ2-containing complexes was slower than that of γ1-complexes, both in the absence and presence of 991. At the 15 min time point, the percentage of AMPK activity remaining was 24 ± 5 versus 45 ± 5% (for the AMPKγ1 complex in the absence or presence of 991, respectively; *n* = 5 ± SEM) and 54 ± 4 versus 70 ± 3% (for AMPKγ2 complex in the absence or presence of 991, respectively; *n* = 5 ± SEM). These results demonstrate that γ2 is more resistant to dephosphorylation by PP2C in cell-free assays. This finding provides a potential mechanism for the increased Thr^172^ phosphorylation in γ2-complexes in response to 991 in cells. Furthermore, this finding may also account for the increased basal level of Thr^172^ phosphorylation in γ2-complexes relative to γ1-complexes that was observed in HEK293T cells.

## Discussion

Our results show that AMPK complexes containing the γ2-isoform have increased Thr^172^ phosphorylation compared with either γ1- or γ3-complexes in response to 991 in human cells. This effect is mediated by the long N-terminal region present in γ2, since a chimeric protein containing the C-terminal region of γ1 fused to the N-terminal region of γ2 mimicked the response of native γ2 to 991. Furthermore, we demonstrate that a potential mechanism for the enhanced phosphorylation of Thr^172^ in γ2 AMPK is a decrease in the rate of dephosphorylation by protein phosphatases. This effect was seen in the absence and presence of 991, suggesting that it is an intrinsic feature of γ2-containing complexes. This would explain the finding that the level of Thr^172^ phosphorylation in γ2-complexes is greater than that of γ1- or γ3-complexes in untreated cells. At present, it is not clear how the N-terminal region of γ2 could interfere with dephosphorylation of Thr^172^. The simplest explanation would be that the long N-terminal extension of γ2 sterically hinders access to Thr^172^ by protein phosphatases. Another possibility would be that the N-terminal region of γ2 interacts with a distinct region of AMPK causing a conformational change within the AMPK complex which leads to increased protection against dephosphorylation. At present, however, there is no molecular structural information for AMPK γ2-containing complexes to support either of these hypotheses. The N-terminal region of γ2a (residues 1–241) shows no obvious sequence similarity to other proteins. Analysis using the PSIPRED web server [40], a secondary protein structure prediction package, reveals that residues 1–220 are predicted to have a high probability of being intrinsically disordered. If this were the case, it would raise many possibilities for how this region might play a role in regulating AMPK activity. For instance, the N-terminal region might bind to other proteins and/or undergo changes in post-translational modification, features commonly associated with intrinsically disordered proteins [41]. It is interesting to note that we have been unsuccessful in all our efforts to express AMPK complexes containing the long isoform of γ2 in bacteria. Furthermore, in a recent publication investigating different AMPK complexes expressed in bacteria, the γ2 complex was not studied [21], although no reason was stated for its omission. It is possible that stable expression of the AMPK complex containing the long isoform of γ2 requires another protein or cofactor, and/or post-translational modification, that is not present in bacterial cells. Further work is required to explore these possibilities. Similarly, we have been unable to express the short form of γ2 (γ2b), or a chimeric protein comprising the short N-terminal sequence present in γ2b fused to the C-terminal core region of γ1, in mammalian cells. It is possible, therefore, that the short N-terminal sequence in γ2b destabilises the AMPK complex in mammalian cells. Two related studies reported protein expression of AMPK complexes containing γ2b in mouse heart [34,42], although the specificity of the anti-AMPKγ2 antibodies used in these studies was not demonstrated convincingly. More recently, evidence for the expression of the long form of γ2 (γ2a) in mouse liver and heart was reported, and in this case, the specificity of the antibody used was demonstrated using tissues from AMPKγ2 knockout mice [43]. Thus, while direct evidence for protein expression of γ2b *in vivo* remains equivocal, it is clear that γ2a protein is expressed *in vivo*.

A recent study investigated the effects of the different γ subunit isoforms on AMPK regulation by nucleotides [8]. In that study, similar to our current study, AMPK complexes were expressed in human cells. Our results complement and add to the previous study by investigating the effects of the different γ-isoforms on AMPK regulation by a pharmacological activator, 991. One potential problem with expressing AMPK in mammalian cells is the possibility that endogenous AMPK subunits might compromise interpretation of the results. To mitigate against this possibility, we generated HEK293T cells is which both the β1- and β2-isoforms were genetically deleted. We chose to delete the β-subunit isoforms as previous studies have shown that the β-subunit plays a fundamental role in binding small-molecule AMPK activators, and that there is a significant difference in binding between AMPK complexes containing either the β1- or β2-isoform [22,24]. The β1β2 dKO cells provide a human cell line that is readily amenable to transfection (transient or stable) that can be used to investigate the regulation of the β-isoforms, including mutant variants of these isoforms, by different compounds. In addition, it is possible that a natural ligand exists that binds to the same site as the pharmacological activators, although no such ligand has been identified to date. The availability of cell models expressing different forms of the β-subunit, e.g. full-length protein versus a C-terminal fragment lacking the CBM required for compound binding, will also provide useful tools in searching for endogenous AMPK activators.

We show that total AMPKα expression, γ1, pThr172 and pACC were almost completely undetectable in the β1β2 dKO cells. A caveat to the present work, however, is that we have been unable to determine directly the effect of β-subunit deletion on endogenous γ2- and γ3-expression. This may reflect the finding that γ1 is the predominant γ-isoform expressed in HEK293 cells [37], and/or could be due to the lack of availability of sensitive antibodies against these isoforms. Nonetheless, the almost complete loss of functional AMPK provides an excellent background in which to express AMPK complexes of defined isoform composition. As part of our validation of the β1β2 dKO cells, we stably reintroduced either β1- or β2-isoforms in order to generate cells expressing exclusively either β1 or β2 and treated them with two different pharmacological AMPK activators, with different β-isoform selectivity. The effect of the activators on phosphorylation of Thr^172^ and ACC correlated extremely well with their β-isoform selectivity determined using purified AMPK complexes. These cells will provide a useful resource for future studies aimed at determining β-isoform selectivity of different AMPK activators in a cellular context.

An important finding that emerged from our study is that Ser^108^ in β2 does not appear to play a role in AMPK activation by 991. This is in contrast with β1, where phosphorylation of Ser^108^ increases 991 binding by 50–100-fold, depending on the nature of the α-isoform present in the complex [24]. In β1, pSer^108^ forms many electrostatic interactions with residues from the α-subunit as well as Asn^111^ in β1 [24]. The sequence surrounding Ser^108^ is well conserved between β1 and β2, although in β2 residue 111 is an aspartate, rather than an asparagine present in β1 [35]. To date, however, there is no high-resolution structural information for AMPKβ2 complexes bound to 991 (or other related pharmacological activators), and hence the molecular basis for the difference in requirement for Ser^108^ phosphorylation between the β-isoforms is not clear. In β1, Ser^108^ is capable of undergoing autophosphorylation [44], although it is not known whether there are other kinases capable of phosphorylating this site. As far as we aware, there are no published data on Ser^108^ phosphorylation of β2, and hence further studies will be required to determine whether Ser^108^ phosphorylation in β2 plays a role in the regulation of AMPK. Although the physiological significance of Ser^108^ phosphorylation for either β1 or β2 remains enigmatic, the finding that it is not required for 991-induced activation of β2-complexes may prove significant for future studies exploring pharmacological regulation of AMPK.

Pharmacological activation of AMPK has attracted significant attention as a potential target for the treatment of metabolic diseases. Many potent AMPK activators have recently been identified, although information regarding their effects in *in vivo* models is currently lacking. Understanding whether there are any substantive differences in pharmacological activation of different AMPK complexes would provide useful complementary information for designing and interpreting subsequent *in vivo* studies. A recent *in vitro* study investigated the effect of A769662, the first direct synthetic AMPK activator to be identified [45], on different AMPK complexes, but this did not include γ2-containing complexes [21]. In that recent study, recombinant AMPK complexes purified from bacteria were used, and no cell-based studies were included. In our current study, we used human cells for expressing AMPK complexes and an important consequence of this approach is that we were able to express complexes containing all three γ-isoforms. Another potentially important advantage gained by using AMPK complexes expressed in human cells is that they are likely to undergo the same post-translational modifications that occur *in vivo* and would be expected to recapitulate more closely the native enzyme. One of the key reasons for carrying out the current study was to investigate whether the different γ-isoforms has an impact on pharmacological activation of AMPK. In addition to demonstrating that 991-induced Thr^172^ phosphorylation is greatest in γ2-containing complexes, we also found that the basal phosphorylation of AMPKγ2 is increased relative to either γ1 or γ3. Despite significant interest in AMPKs, the physiological relevance of the different AMPKγ isoforms is poorly understood. There is evidence for differences in tissue expression of the different isoforms. For example, expression of the γ3-isoform is restricted to skeletal muscle [36,39], which might suggest tissue-specific roles for certain AMPK complexes. However, direct experimental evidence to support this hypothesis is lacking. Does our finding that γ2-containing AMPK complexes have higher basal activity than the other γ-isoforms have any physiological significance? One possibility is that in human heart, where γ2-expression is highest [36], the increased basal activity of AMPKγ2 complexes is required to maintain cardiac-specific functions. In this respect, it is interesting that a previous study reported that ADP was as effective as AMP in protecting γ2-complexes against dephosphorylation, whereas AMP was much more effective compared with ADP in protecting either γ1- or γ3-complexes [8]. This differential regulation could perhaps be important during periods of altered cardiac energetics which result in changes in adenine nucleotide levels.

The finding that 991-induced Thr^172^ phosphorylation is greatest in γ2-containing complexes may have implications for the therapeutic potential of long-term pharmacological activation of AMPK. Gain-of-function mutations in γ2 lead to a cluster of cardiac abnormalities in humans. Clearly, a major concern in targeting AMPK for activation is the possibility of developing phenotypes that overlap with those resulting from the γ2 mutations. In this regard, the increased sensitivity of γ2-containing complexes could add to this potential risk. Many strategies could be envisaged to mitigate against this potential adverse effect. For instance, the beneficial effects of AMPK activation on metabolism are likely to be mediated by non-cardiac tissue. It may be possible to design compounds with tissue-specific delivery, and hence prevent accumulation in the heart. A greater understanding of the isoform composition of AMPK complexes in human heart might also provide nuanced strategies for pharmacological targeting. If, for example, γ2 is largely associated with β2 in the heart, development of β1-selective activators would greatly reduce activation of the γ2-complexes. The identification of highly selective β1-activators [22] is an encouraging step in this approach.

## Abbreviations

ACC: acetyl-CoA carboxylase
AMPK: AMP-activated protein kinase
ANOVA: analysis of variance
CBM: carbohydrate binding module
CBS: cystathionine-β-synthase
CRISPR: clustered regularly interspaced short palindromic repeats
dKO: double knockout
DMEM: Dulbecco's modified Eagle's medium
DMSO: dimethyl sulphoxide
HEPES: 4-(2-hydroxyethyl)-1-piperazineethanesulfonic acid
LKB1: liver kinase B1
PAGE: polyacrylamide gel electrophoresis
PEI: polyethylenimine
PP: protein phosphatase
SAMS: the synthetic peptide HMRSAMSGLHLVKRR
SDS: sodium dodecyl sulphate
